# Supplementary material for: Spin noise gradient echoes
Source: Magn Reson (Gott). 2021 Nov 19;2(2):827–34. doi: 10.5194/mr-2-827-2021 (PMC10539801; doi:10.5194/mr-2-827-2021)
Supplement: The supplement related to this article is available online at: https://doi.org/10.5194/mr-2-827-2021-supplement. [file mr-2-827-supplement.pdf]

Supplement of Magn. Reson., 2, 827–834, 2021
https://doi.org/10.5194/mr-2-827-2021-supplement
© Author(s) 2021. CC BY 4.0 License.

Supplement of

Spin noise gradient echoes

Victor V. Rodin et al.

Correspondence to: Norbert Müller (norbert.mueller@jku.at)

The copyright of individual parts of the supplement might differ from the article licence.

1 Simulations of spin-noise gradient echo

To assess the effect of SNGE, we perform simulations using Mathematica (Wolfram Research Inc., 2012) based on the Bloch equations (Bloch, 1946), and neglecting any effect of radiation damping. This last assumption corresponds to the used experimental conditions where the inhomogeneous broadening by the gradients exceeds the radiation-damping rate. In these simulations, spin noise originates from a series of random excitation events of random timing, random phase and a random small flip angle supposed to be below $0.01°$. For a single noise event, the evolution of magnetization in the rotating reference frame is simulated by assuming a weak randomized initial transverse magnetization M_0 starting at a random time τ within the first gradient G_1. Using z as the position coordinate along the gradient axis, we get for a single isochromat with offset 0 at the end of G_1:

$$\mathrm{I}(G_1) = M_0 e^{-i\gamma z G_1 \delta_1} \quad \text{(S1)}$$

In the case of a distribution of components along z, it is necessary to integrate:

$$I_l(G_1) = \int_{-l/2}^{l/2} I(G_1)dz \quad \text{(S2)}$$

For the sample with length l, it results in:

$$I_l(G_1) = M_0 \frac{\sin(0.5\gamma G_1 \delta_1 l)}{0.5\gamma G_1 \delta_1} \quad \text{(S3)}$$

Then, the evolution during the second gradient leads further to:

$$\mathrm{I}(G_2) = M_0 e^{-i\gamma z(G_1\delta_1 + G_2\delta_2)} \quad \text{(S4)}$$

Here we assume the delays Δ and ε (Fig. 1) to be negligibly short. After integration over the sample, we get the expression:

$$I_l(G_2) = \int_{-l/2}^{l/2} I(G_2)dz = M_0 \frac{\sin[0.5\gamma(G_1\delta_1 + G_2\delta_2)l]}{0.5\gamma(G_1\delta_1 + G_2\delta_2)} \quad \text{(S5)}$$

By numeric summation over 100 uniformly random distributed (with respect to starting times, phases, and amplitudes) initial magnetizations M_0, we obtain the curves shown in Fig. S1. The case including T_2 relaxation is described by Eq. (S6):

$$I_l(G_2, T_2) = \int_{-l/2}^{l/2} I(G_2)dz = M_0 \frac{\sin[0.5\gamma(G_1\delta_1 + G_2\delta_2)l]}{0.5\gamma(G_1\delta_1 + G_2\delta_2)} e^{-\frac{\delta_1+\delta_2}{T_2}} \quad \text{(S6)}$$

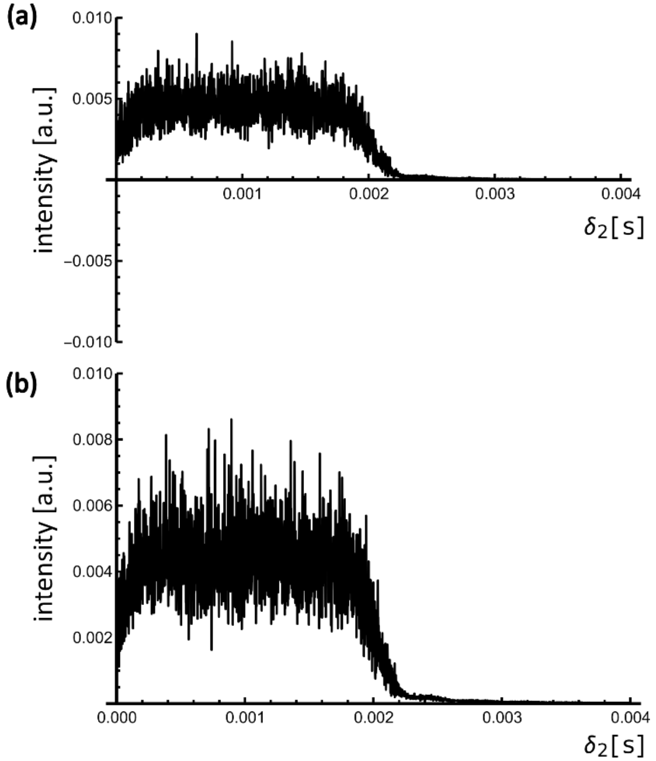

Figure S1. Numerical simulation of the time course of refocused spin noise during the second gradient of a gradient echo sequence, using Eq. S6; (a) neglecting transverse relaxation and diffusion, (b) including transverse relaxation $T_2 = 100$ ms. Experimental parameters corresponding to Fig 1(b) are used in the simulation: $G_1 = -3.2\,\frac{\mathrm{mT}}{\mathrm{m}}$; $G_2 = 3.2\,\frac{\mathrm{mT}}{\mathrm{m}}$; $\gamma = 26.75 \times 10^7\,\frac{\mathrm{rad}}{\mathrm{sT}}$; M_0=1; l = 2 cm. No additional non-spin random noise was added. It would exceed the echo amplitude without extensive averaging.

These simulations assume that for small radiation damping contribution (gradient broadening of the linewidth larger than the initial linewidth due to transverse relaxation and radiation damping), spin noise can be envisaged as a series of spontaneous excitation events of random phase. Because of creating a series of random coherences, they cannot be applied to the cases with weaker gradients or for strong polarization situations, as it was the case in (Desvaux, 2013; Pöschko et al., 2017). After these excitations, the individual coherences can be defocused and refocused by field-gradient pulses, as usual. Fig. S1 only shows the contribution from the refocused noise initiated during δ_1. The signal envelope indicates an echo center at the point where $\delta_2 \leq \frac{1}{2}\delta_1$ in the presence of transverse relaxation (Fig. S1(b)). The location of the maximum gradient echo is typically that observed with coherent excitation ($\frac{\delta_2}{\delta_1} \approx 0.5$).

As a consequence of these simulations, the noise power amplitude of the $I(+)$ experiments (Fig. 1) is due to transverse coherences excited during the second gradient only. However, the additional contributions observed in the $I(-)$ experiments originate from events during first gradient with length of δ_1 refocused by the second gradient during δ_2. A quantum mechanical description of spin-noise dynamics, based on the random pure states model in the work of Field and Bain (Field and Bain 2010 and Field and Bain 2013) would be a promising next step in understanding the dynamics of SNGE experiments.

2 Molecular diffusion characterization

Figure S2 presents the results of SNGE attenuation vs gradient values for a mixture composed of acetone and benzene in 1:1 concentration. The associated spectra are reported in Fig. 4.

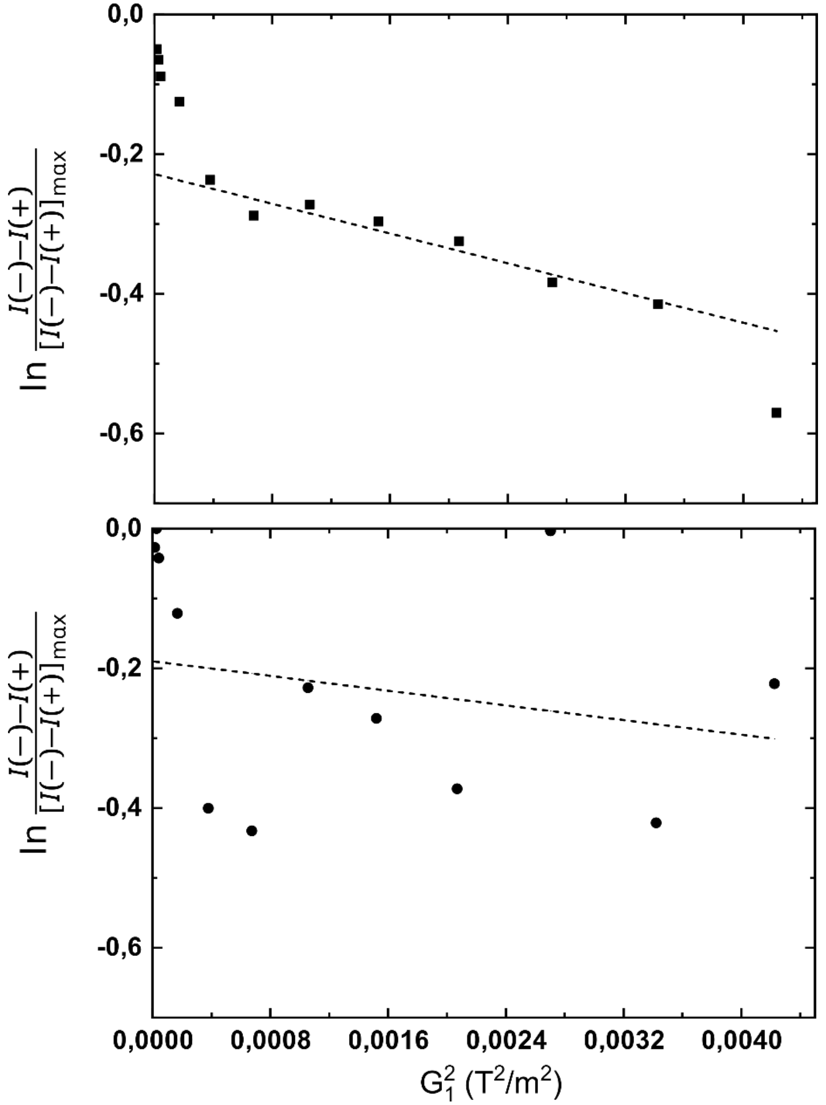

Figure S2. SNGE attenuations vs gradient increase for separate signals of acetone (top) and benzene (bottom) in a 1:1 mixture of acetone and benzene (with 10% of acetone-d6 for locking) recorded according to the scheme of Fig. 3. Experimental spectra are reported in Fig. 4.

As in Fig. 5, Fig. S2 reveals a correct linear behaviour for not too small G_1^2 values. This is particularly true for acetone, while for benzene which molecular diffusion coefficient is smaller, more scattered measurements are observed. From this SNGE attenuation vs G_1^2 experiment (Fig. S2), an estimation of ratio of slope for acetone component in this mixture to that for benzene component results can be determined. This ratio is comparable to the independent determination of diffusion coefficients in pure solvents (acetone and benzene) measured with a SNGE NMR-diffusion experiment (Fig. 3) with a small RF pulse of $0.2\mu s$ added ($2.4 \cdot 10^{-9} \frac{\mathrm{m}^2}{\mathrm{s}}$ for benzene and $4.58 \cdot 10^{-9} \frac{\mathrm{m}^2}{\mathrm{s}}$ for acetone). These self-diffusion coefficients agree well with literature values of benzene and acetone as obtained by canonical RF NMR spin-echo experiments. (Ertl, and Dullien, 1973) This experiment (Fig. S2) illustrates that in two-component mixture with different chemical shifts it is possible to acquire SNGE attenuation data (Fig. 4) and to extract SNGE attenuation curves separately for each diffusion component. So, qualitative comparison of how SNGE signal is attenuated in diffusion experiment in mixtures with differently diffusing (and chemically shifted) molecules. The issue resides in obtaining enough measurements in the range of gradient values (i.e., in such linear range of G_1^2) where mostly diffusion effect should result in measurable decrease in echo intensity (Fig. 4 and S2). However, experimental points (at the largest gradient values) have been measured at $70 \frac{\mathrm{mT}}{\mathrm{m}}$ to $75 \frac{\mathrm{mT}}{\mathrm{m}}$ (Fig. 5 and Fig. S2). This is still not enough to get data set for calculation of diffusion coefficient according to Eq. (3). We could not increase measurable gradient values to carry out reliable SNGE experiment at gradients higher than $75 \frac{\mathrm{mT}}{\mathrm{m}}$. So, implementation of diffusion experiment limited by the fast gradient duty cycle – this is too fast for our high-resolution probe. Therefore, a search of additional experimental facilities can result in new advantages for realization of spin-noise gradient echo measurements.

3 Molecular diffusion and relaxation measurements

Figure S3 illustrates how 3 gradient pulses scheme (Fig. 3) produces SNGE data sets in 1:1 acetone-benzene mixture vs Δ.

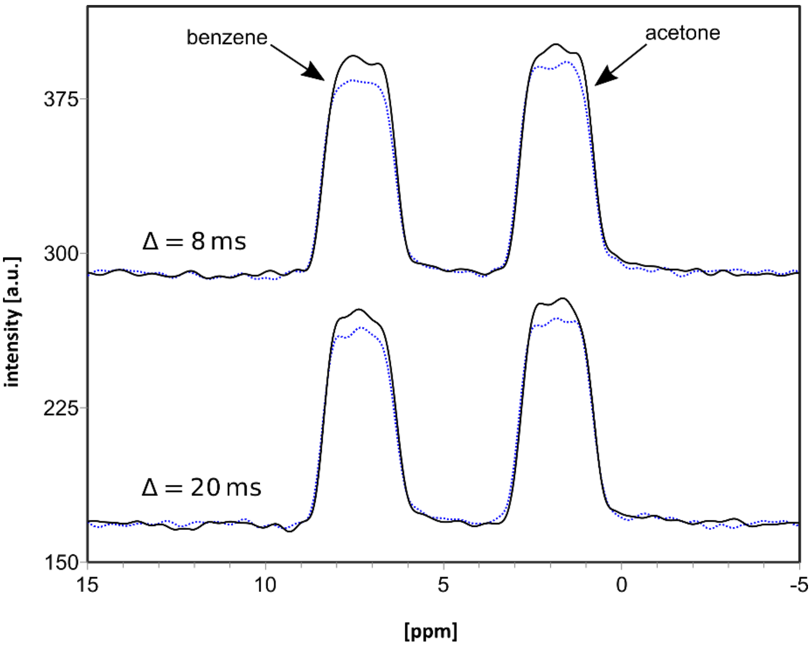

Figure S3. SNGE experiments as a function of Δ between G_1 and G_2 with a triple gradient sequence (Fig. 3) on a 1:1 mixture of acetone and benzene (with 10% of acetone-d6 for locking). The respective lower traces correspond to the $I(+)$ sub-experiment, the higher ones are to the $I(-)$ measurements. SNGE spectra are presented for 2 values of delay increasing from top to bottom: 8 ms, 20 ms. Chemical shifts: 2.1 ppm (acetone); 7.2 ppm (benzene). (Experimental conditions as in Fig. 6)

Data presented show that in the mixture of two pure solvents with different chemical shifts of their molecules (for instance, benzene and acetone) SNGE attenuation (i.e., normalized difference between z-profile signals in $I(-)$ and $I(+)$ spin-noise experiments vs gradient history) can be presented separately with measurable relaxation constants. An estimation of relaxation constant for separate component (benzene) in mixture appears similar to that measured in SNGE experiment on pure solvent (benzene) (Fig. 6 and Fig. S4).

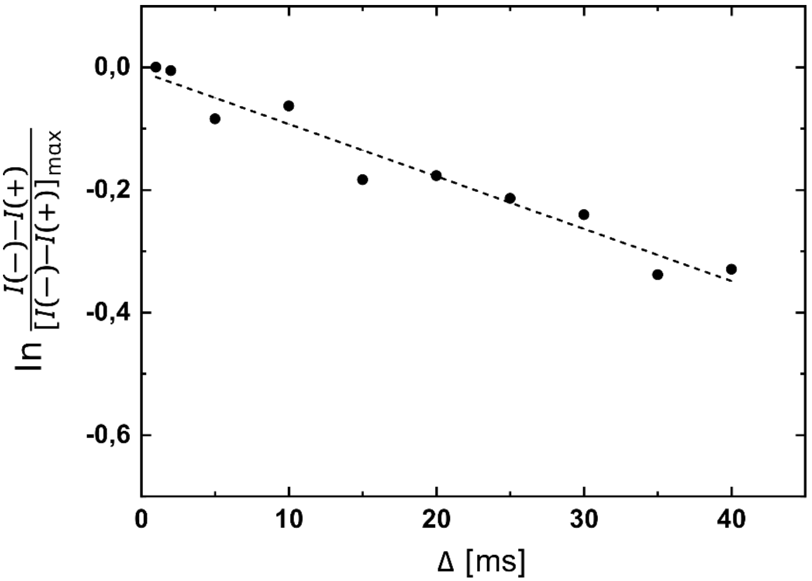

Figure S4. The $I(+)$ and $I(-)$ ^{1}H SNGE experiments on pure benzene for different delays Δ between G_1 and G_2 with triple gradient sequence. Relaxation constant for benzene $T_2^* = 87$ms to 93 ms. ($G_1 = 63\,\frac{\mathrm{mT}}{\mathrm{m}}$, $G_2 = 123\,\frac{\mathrm{mT}}{\mathrm{m}}$, $G_3 = 1.6\,\frac{\mathrm{mT}}{\mathrm{m}}$, $\delta_1 = 2$ ms, $\delta_2 = 1$ ms, gradient stabilization delay $\epsilon = 0.05$ ms, acquisition time $t_{aq} = 3.69$ ms).

For comparison we have determined the apparent T_2* in a continuously recorded spin-noise spectrum without any gradients or pulses (by the usual protocol of ref (Nausner et al., 2009), which is shown in Fig. S5. From these data T_2^* values of 8.5 ms and 8.0 ms have been determined by a Lorentzian line-shape fit.

The difference in the relaxation times (T_2^*) determined from the spin noise line shape and the one from spin noise amplitude decay in the inter-gradient delay is ascribed to different radiation damping times which are subject to influence of preamplifier feedback (see main text) (Pöschko et al., 2017)

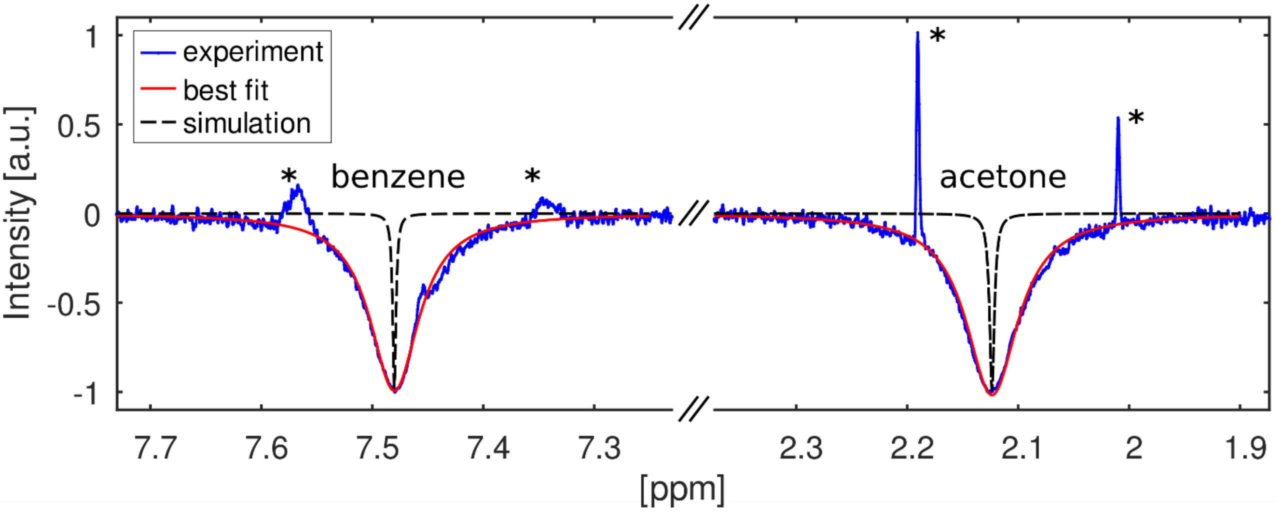

Figure S5. (blue) Spin noise spectrum of a 1:1 mixture of benzene and acetone (as in Figure 6), but recorded without any gradients or pulses. (red) Lorentzian best fit of experimental peaks amounting to $FWHM$ = 37.6Hz / 39.8Hz and hence $T_2^*= 1/(\pi \cdot FWHM)$ = 8.5ms / 8.0ms for benzene / acetone respectively. (black) Simulated Lorentzian resonance peaks assuming T_2^*'s as determined from three-gradient experiments (c.f. Fig. 3) (92ms / 62.5ms for benzene/acetone respectively). The asterisks (*) indicate signals of ^{13}C satellites as described in (Pöschko et al., 2017)

References

Bloch, F.: Nuclear induction, Phys. Rev., 70(7–8), 460–474, doi:10.1103/PhysRev.70.460, 1946.

Desvaux, H.: Non-linear liquid-state NMR, Prog. Nucl. Magn. Reson. Spectrosc., 70, 50–71, doi:10.1016/j.pnmrs.2012.11.001, 2013.

Ertl, H. and Dullien, F.A.L.: Self-diffusion and viscosity of some liquids as a function of temperature. AIChE J., 19, 1215–1223. doi:10.1002/aic.690190619, 1973.

Field, T. R. and Bain, A. D.: Origins of Spin Noise , Appl. Magn. Reson. 38, 167, doi: 10.1007/s00723-009-0107-2, 2010.

Field, T. R. and Bain, A. D.: A dynamical theory of spin relaxation, Phys. Rev., E 87 022110, doi: 10.1103/PhysRevE.87.022110, 2013.

Pöschko, M. T., Rodin, V. V., Schlagnitweit, J., Müller, N. and Desvaux, H.: Nonlinear detection of secondary isotopic chemical shifts in NMR through spin noise, Nat. Commun., 8(1), 1–9, doi:10.1038/ncomms13914, 2017.

Wolfram Research Inc.: Mathematica, Version 9, Champaign, Illinois. [online] Available from: https://www.wolfram.com/mathematica, 2012.
